# Correction to: Comparison of whole-genome bisulfite sequencing library preparation strategies identifies sources of biases affecting DNA methylation data

**DOI:** 10.1186/s13059-019-1656-9

**Published:** 2019-02-22

**Authors:** Nelly Olova, Felix Krueger, Simon Andrews, David Oxley, Rebecca V. Berrens, Miguel R. Branco, Wolf Reik

**Affiliations:** 1 Epigenetics Programme, The Babraham Institute, Cambridge, CB22 3AT UK; 2 Bioinformatics Group, The Babraham Institute, Cambridge, CB22 3AT UK; 3 Mass Spectrometry Facility, The Babraham Institute, Cambridge, CB22 3AT UK; 4 Blizard Institute, Barts and The London School of Medicine and Dentistry, Queen Mary University of London, E1 2AT, London, UK; 5 Wellcome Trust Sanger Institute, Hinxton, CB10 1SA UK; 6 Centre for Trophoblast Research, University of Cambridge, Cambridge, CB2 3EG UK; 7 Present address: MRC Human Genetics Unit, MRC Institute of Genetics and Molecular Medicine, University of Edinburgh, Edinburgh, EH4 2XU UK

**Correction to: Genome Biology (2018) 19:33.**

**https://doi.org/10.1186/s13059-018-1408-2**

Following publication of the original article [1], it was reported that the incorrect “Additional file 3” was published. The correct additional file is given below.

## Additional file

Additional file 3: An Excel spreadsheet with reference genome compositions. (XLSX 19 kb)
