# Supplementary material for: Comparison of whole-genome bisulfite sequencing library preparation strategies identifies sources of biases affecting DNA methylation data
Source: Genome Biol. 2018 Mar 15;19:33. doi: 10.1186/s13059-018-1408-2 (PMC5856372; doi:10.1186/s13059-018-1408-2)
Supplement: Supplementary file 2 — A PDF file with supplementary Tables S1–S3 and supplementary Figures S1–S10. (PDF 11655 kb) [file 13059_2018_1408_MOESM2_ESM.pdf]

Table S1 M13-derived double stranded (ds) DNA fragments with variable cytosine content used for the BS conversion and BS degradation analyses.

Name	Length	Sequence 5’ > 3’ (forward strand only)	Context: ds
**C-poor** **(15.7%C)**	201 bp	ccagacgcgaattatttttgatggcgttcctattggttaaaaaa<br>tgagctgatttaacaaaaatttaatgcgaattttaacaaaatat<br>taacgtttacaatttaaatatttgcttatacaatcttcctgttt<br>ttggggcttttctgattatcaaccggggtacatatgattgacat<br>gctagttttacgattaccgttcatc	26 % CG<br>35 % CA<br>18 % CT<br>21 % CC
**C-rich** **(30%C)**	123 bp	ggcgttacccaacttaatcgccttgcagcacatccccctttcgc<br>cagctggcgtaatagcgaagaggcccgcaccgatcgcccttccc<br>aacagttgcgcagcctgaatggcgaatggcgcttt	30 % CG<br>21 % CA<br>19 % CT<br>30 % CC

Table S2 Cytosine content of mouse major satellite consensus repeat and mtDNA.

Sequence	Length (bp)	C forward	C reverse	% C forward	% C reverse
Major satellite	234	32	54	13.68	23.08
mtDNA NCBIM37	16299	3976	2013	24.39	12.35
mtDNA GRCm38	16299	3976	2013	24.39	12.35
mtDNA GRCh37	16571	5192	2180	31.33	13.16
mtDNA GRCh38	16569	5181	2169	31.27	13.09

Table S3 Oligonucleotides used in the amplification and cloning of M13 and the major satellite repeat.

**DNA primers**		
**Region**	**Forward primer**	**Reverse primer**
**M13 2kb**	ATTTCCATGAGCGTTTTTCC	GCAAGGCAAAGAATTAGCAA
**M13 C-poor**	CCAGACGCGAATTATTTTTG	GATGAACGGTAATCGTAAAACTAGC
**M13 C-rich**	GGCGTTACCCAACTTAATCG	AAAGCGCCATTCGCCATT
**MSatFor BS**	AAATGAGAAATATATATTTTAGGA	CAAATAAATATTTCTCATTTTCC
**MSatRev BS**	AAATAAAAAATACACACTTT	GTTAAGTGGATGTTTTTTATT
**Sequencing primers**		
**Region**	**Forward primer**	**Reverse primer**
**M13 (F-40 & R)**	GTTTTCCCAGTCACGAC	CAGGAAACAGCTATGACC
**pQE (FOR-REV)**	CCCGAAAAGTGCCACCTG	GGTCATTACTGGAGTCTTG
**Cloning primers**		
**AttB-M.MpeI F**	GGGGACAAGTTTGTACAAAAAAGCAGGCTTCGGATCCGCCACCATGGATAGCAACAAGGACAAGA	
**AttB-M.MpeI R**	GGGGACCACTTTGTACAAGAAAGCTGGGTCGAATTCTCAGTGGTGGTGGTGGTGGTGCTCG	

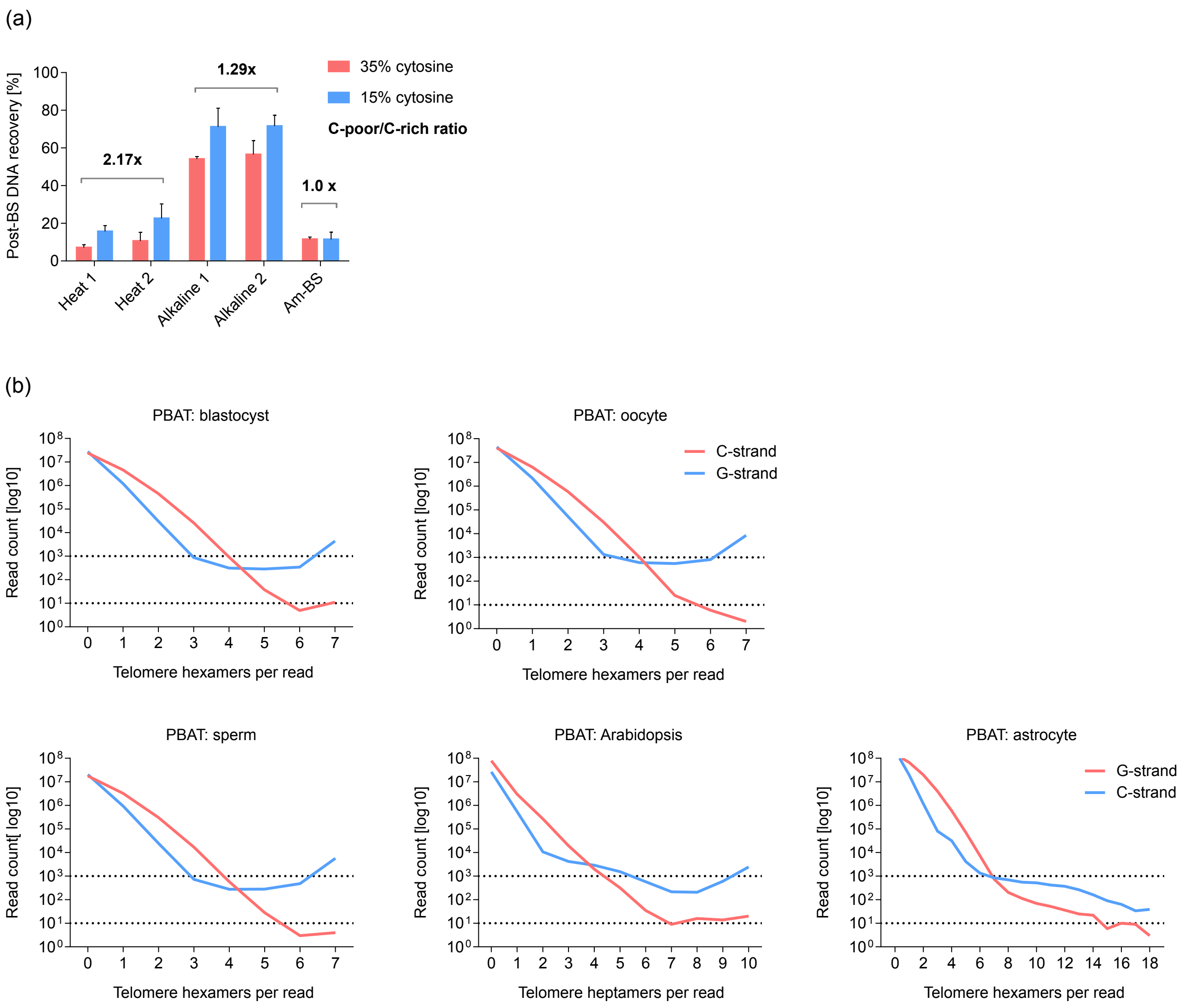

Figure S1. Effect of DNA fragmentation on base composition of BS-treated DNA. (**a**) Post-bisulfite recovery of C-rich and C-poor DNA fragments treated with five different BS-conversion protocols. Fragment sequences originate from the M13 phage sequence (Additional File 3: Table S1). Ratios with fold-change in recovery between C-rich and C-poor are pointed in bold above horizontal brackets. Error bars represent s.e.m. (**b**) Telomere repeat count per read in different amplification-free PBAT WGBS. G-strand tandems ([TTAGGG]$_n$) were quantitated separately from unconverted and BS converted C-strand tandems ([CCCTAA]$_n$ and [TTTTAA]$_n$ respectively) in order to assess the fragmentation of the C-strand with increase of tandem count and cytosine content. Each plot represents a single dataset. C-strand reads containing less than four to six tandems are genuine [TTTTAA]$_n$ repeats of non-telomere origin (results not shown, see Methods).

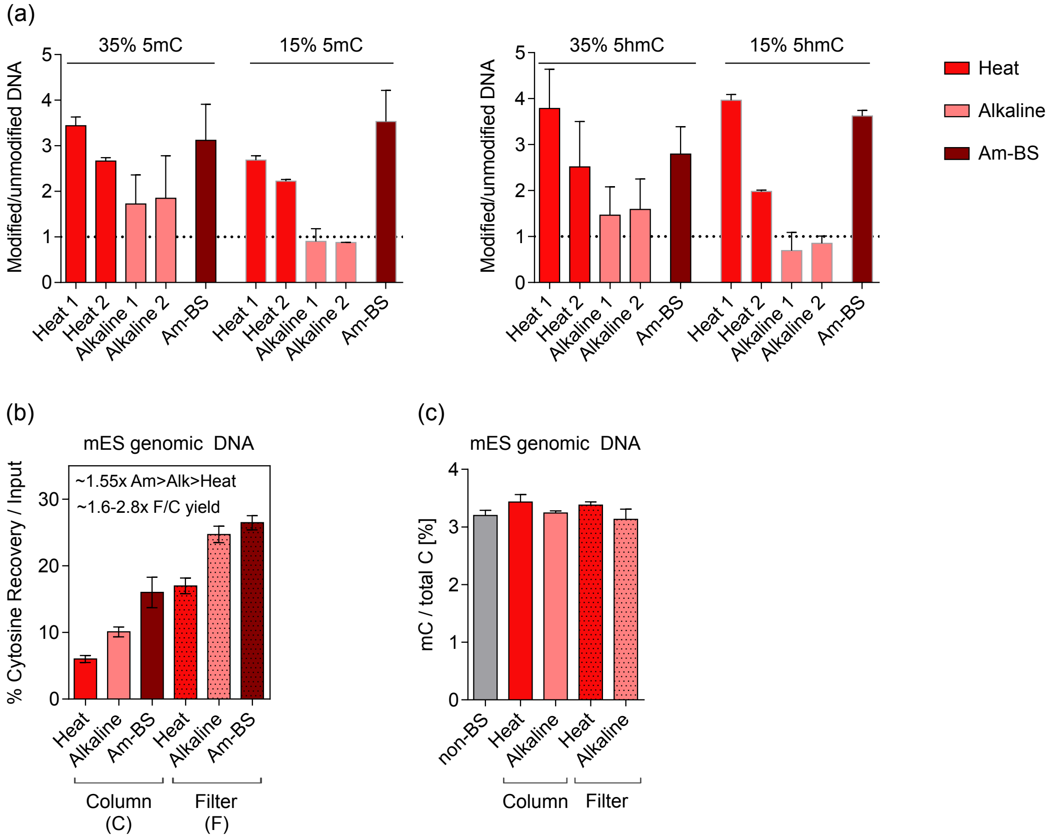

Figure S2. Effect of modification status and DNA clean-up methods on BS-induced DNA fragmentation. (**a**) Fold-difference in the post-bisulfite recovery of fully methylated (left) or hydroxy-methylated (right) C-rich and C-poor DNA fragments, treated with different BS conversion protocols. The fold-difference is against the recovery of unmethylated control fragments for each method. (**b**) Post-BS conversion recovery of cytosine in mouse ESCs gDNA after two different DNA clean-up procedures-spin columns and accompanying reagents from the BS conversion kits and 30k molecular filters with reagents used in the TrueMethyl kit v1. Yield ratios between the different conversion methods or clean-up procedure are indicated in the figure. (**c**) LC/MS measurement of total genomic 5mC levels in mouse ESC gDNA purified by spin columns or molecular filters. Error bars represent s.d.

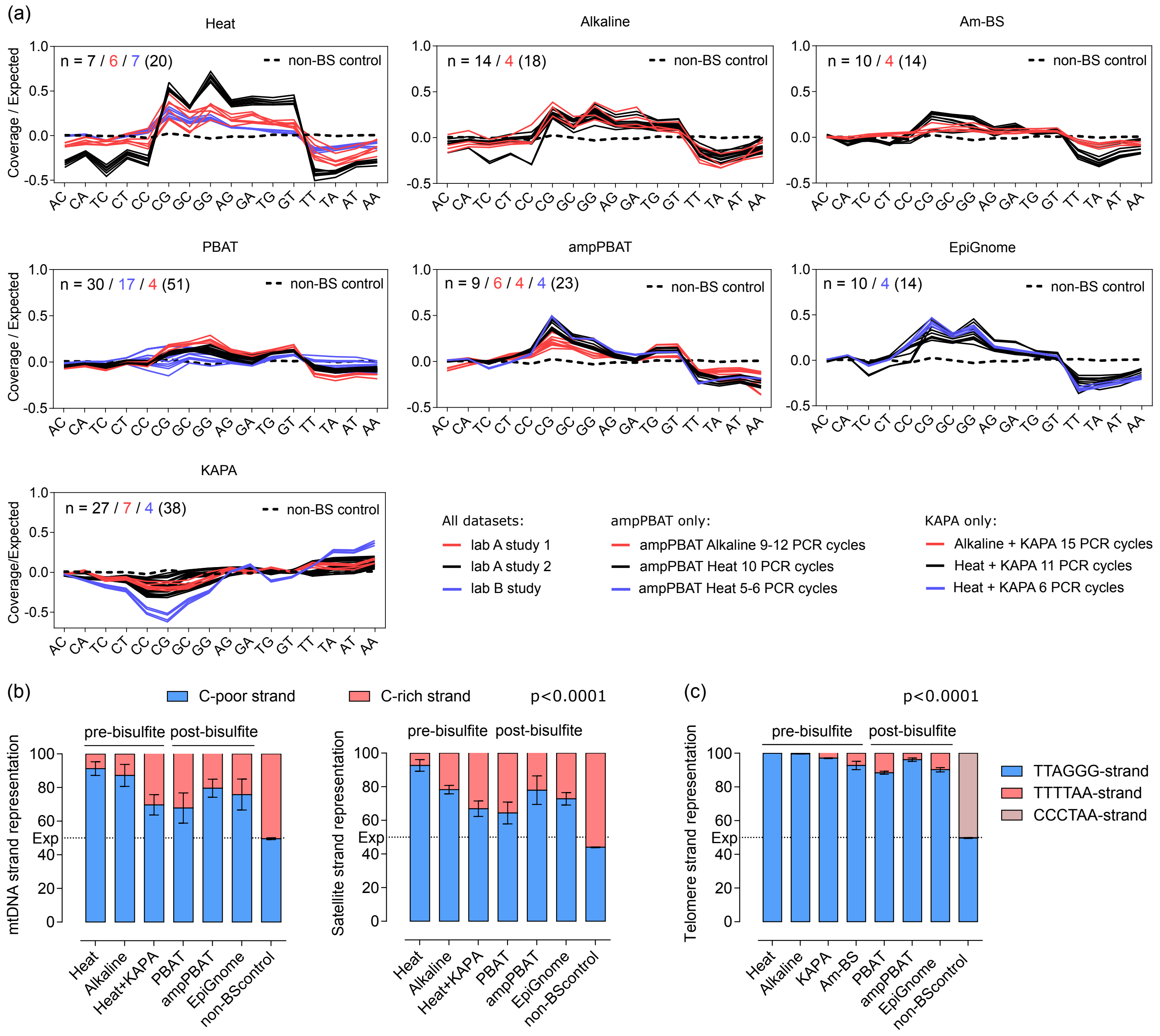

Figure S3. Effect of polymerase bias and library preparation protocols on genomic base composition. (**a**) Dinucleotide coverage in WGBS datasets from different studies prepared with different protocols and compared to a non-BS control. The coverage is expressed as log2 difference from the genomic expected value. Each line corresponds to a single library/sequencing run and each colour represents a separate study. The number of analysed libraries is indicated for each study, with matched colour coding to the lines. Details on study, source laboratory and species can be found in Additional File 1. (**b**) Asymmetric strand coverage of the mouse major satellite and mtDNA in WGBS datasets generated with different library preparation protocols and compared to a non-BS control. The C-poor strands contain 12% (mtDNA) or 14% (major satellite) cytosines, and the C-rich - 24% (mtDNA) and 23% (major satellite) cytosines. The expected unbiased genomic ratio is presented with a dashed line at 50%. (**c**) Coverage of telomere strands in a set of publically available BS-seq datasets and a non-BS control. The G-strand ($[TTAGGG]_n$) and the complementary C-strand ($[CCCTAA]_n$) were mapped separately as well as aBS converted T-version of the C-strand ($[TTTTAA]_n$). Error bars represent s.d. All methods in b) and c) show highly significant difference to the non-BS control ($p<0.0001$) according to a 1-way ANOVA with Bonferroni's multiple comparisons test. The number of compared datasets in each test and method are listed in Additional File 1.

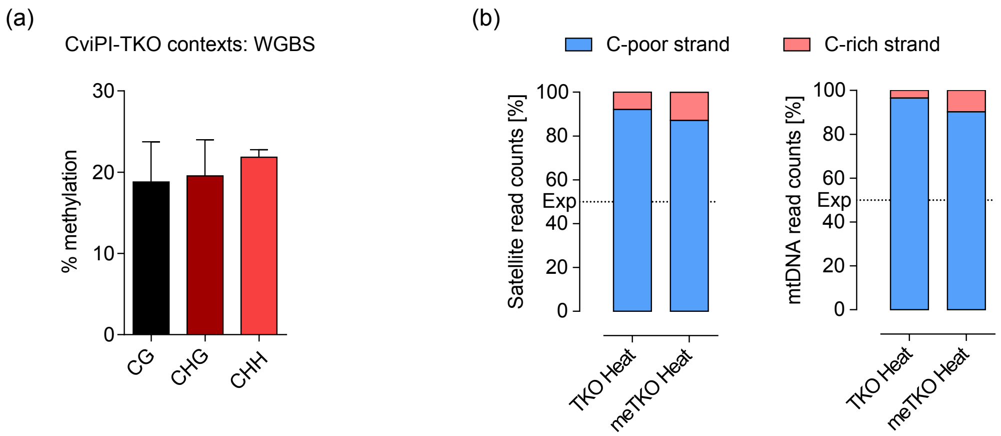

Figure S4. Effect of DNA modifications on WGBS biases. (**a**) Methylation in the different cytosine contexts in the in vitro M.CviPI-methylated TKO DNA. Error bars represent s. d. (**b**) Changes in the proportions of asymmetric strand coverage of the mouse major satellite and mtDNA in an in vitro M.CviPI-methylated TKO WGBS dataset against the unmethylated TKO control.

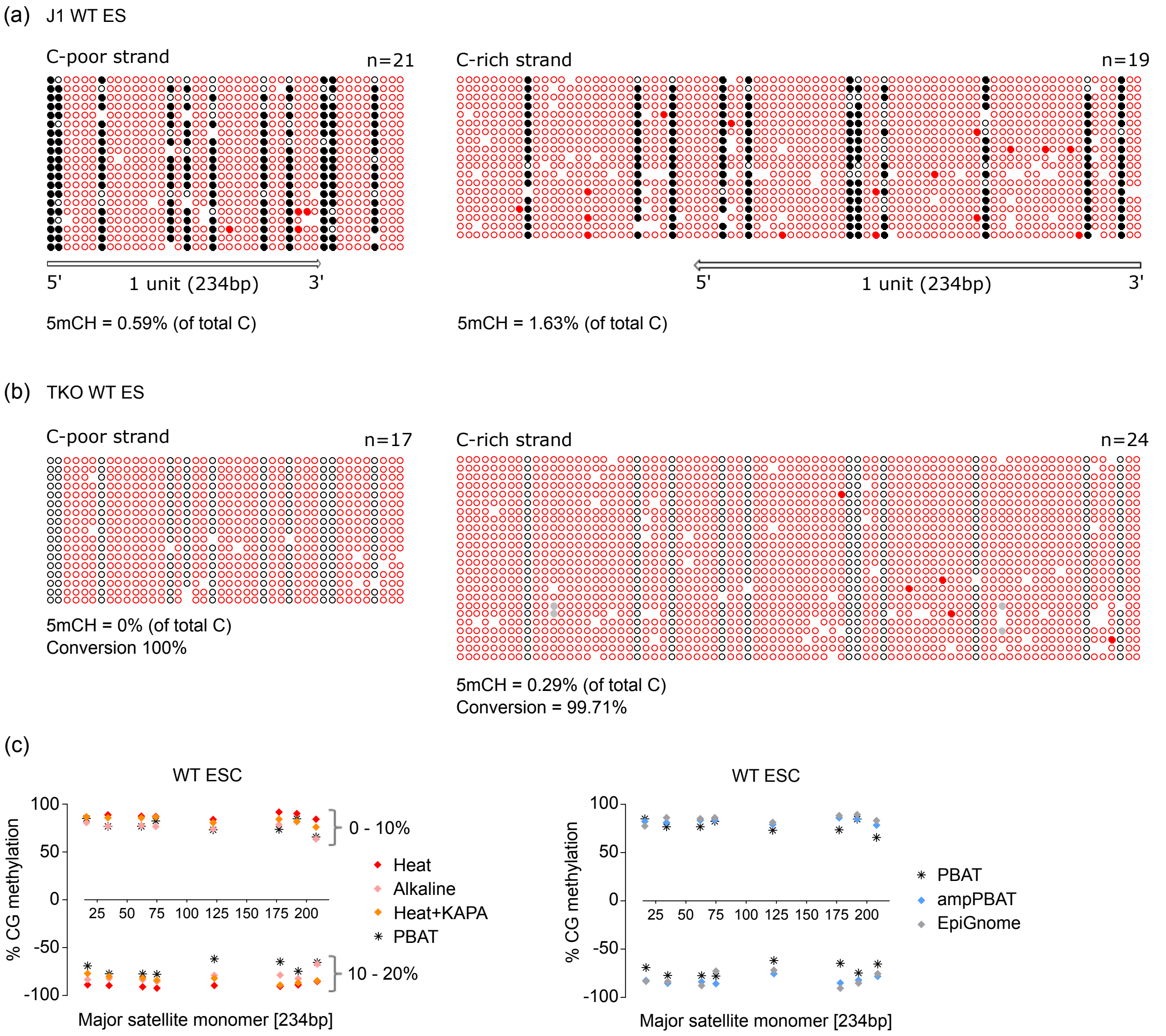

Figure S5. Validation of non-CG context methylation in the mouse major satellite repeat. (**a**,**b**) Classical targeted BS sequencing of the major satellite repeat from (**a**) WT J1 and (**b**) the unmethylated TKO mESC lines. The consensus sequence is 234bp long, usually repeated in tandem in the pericentric region of mouse chromosomes; the sequenced region is longer than one length of one consensus repeat. Shown are the calculated mCH values in the J1 line and the conversion rate of the TKO line. (**c**) mCG methylation of the major satellite consensus, measured by different WGBS protocols. Positive y-axis values represent the top strand and negative – the bottom strand. The absolute value discrepancy between PBAT and 'Heat' protocols is shown: 10-20% for the C-rich reverse strand and 1-10% for the C-poor forward strand.

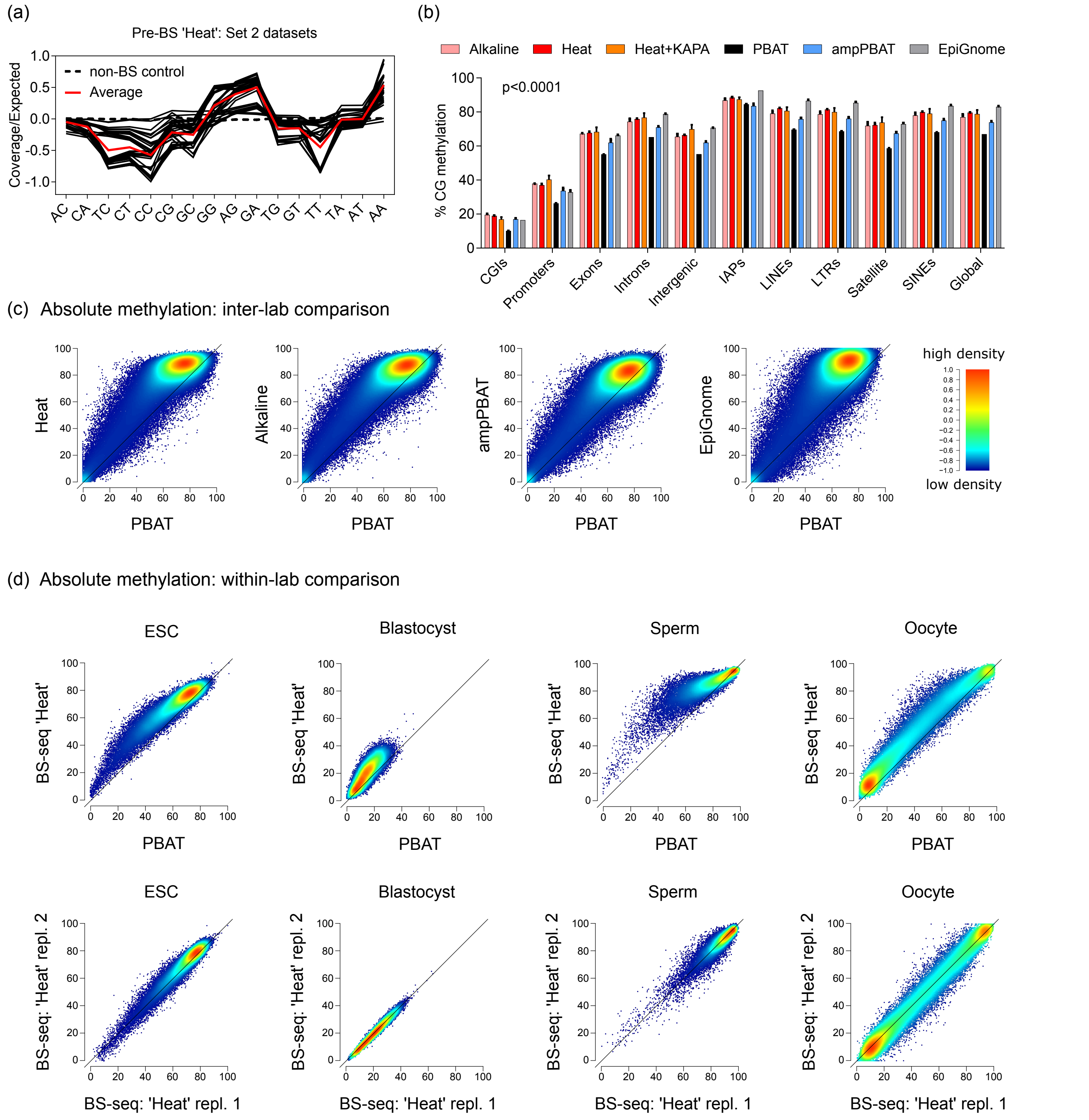

Figure S6. Comparison of methylation quantitation between WGBS datasets. (**a**) Dinucleotide coverage of individual BS-seq datasets (black lines) and averaged (red line) from Kobayashi et al. (2012). The coverage is expressed as log2 difference from the genomic expected value. (**b**) CG methylation values for a selection of standard genomic features in mESCs. Statistical analysis was performed with a two-way ANOVA for all methods in all features against the PBAT values, where p<0.0001 bars indicate s.d. (**c**) Scatter-plot comparison of CG genomic methylation (%) in mESCs between the amplification-free PBAT and the amplified WGBS protocols (see Additional File 1 for details on datasets). Am-BS was not included in this analysis due to lack of appropriate mESC dataset. (**d**) Scatter-plot comparison of the amplification-free PBAT approach and 'Heat' BS-seq performed by Kobayashi et al. (2012) for four biological samples. The colour density legend is the same as for (c).

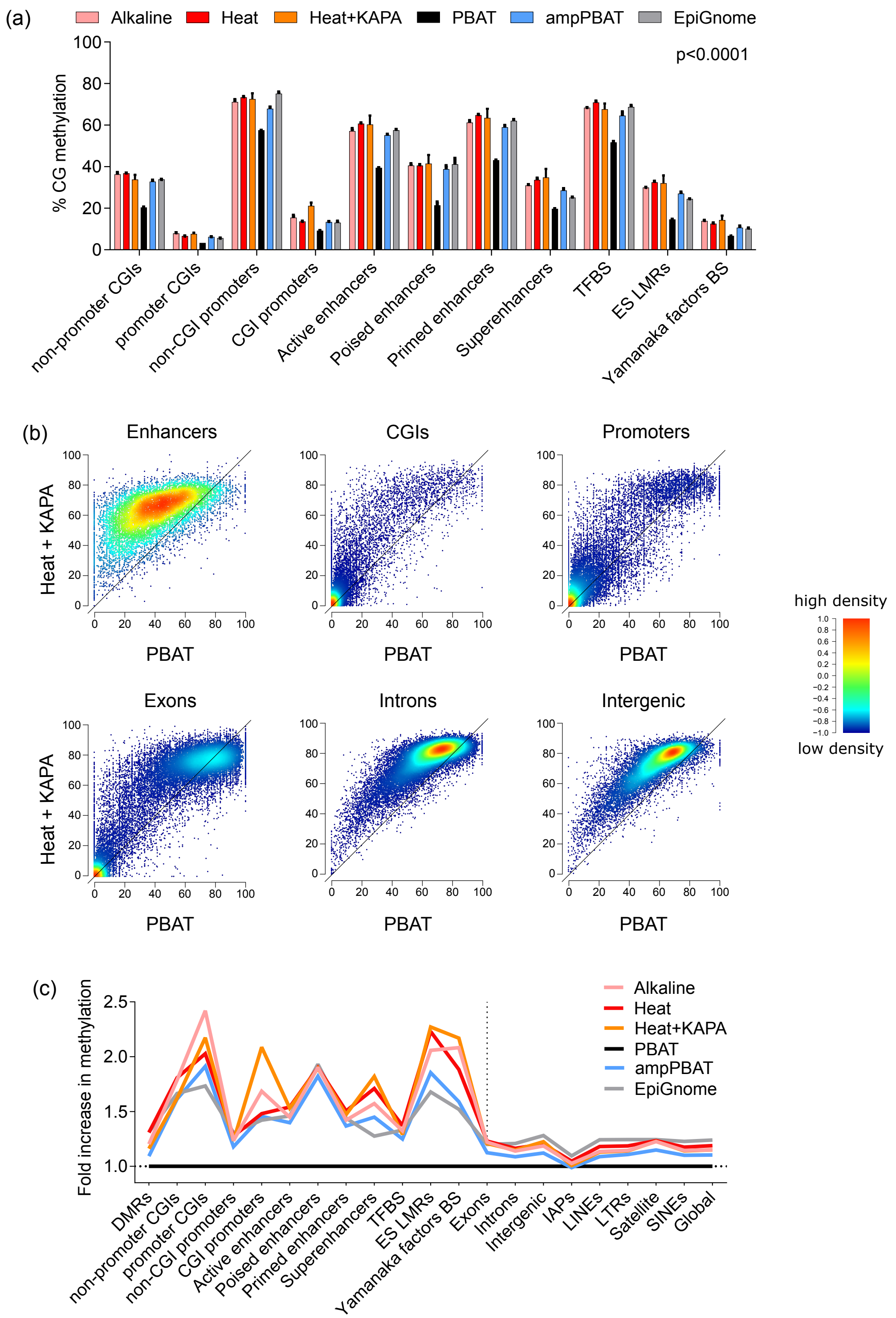

Figure S7. Effect of WGBS protocol on 5mC estimation in intermediately methylated regions. (**a**) CG methylation values for a selection of regulatory genomic features. Statistical analysis was performed with two-way ANOVA for all methods in all features against the PBAT values, where $p < 0.0001$ error bars indicate s.d. (**b**) Direct comparison of CG methylation values between the amplification-free PBAT and the 'Heat+KAPA' BS-seq. (**c**) Higher proportional difference in CG methylation values in regulatory than in other genomic regions (separated by a vertical dotted line). LMRs = Low Methylated Regions, TFBS = Transcription Factor Binding Sites.

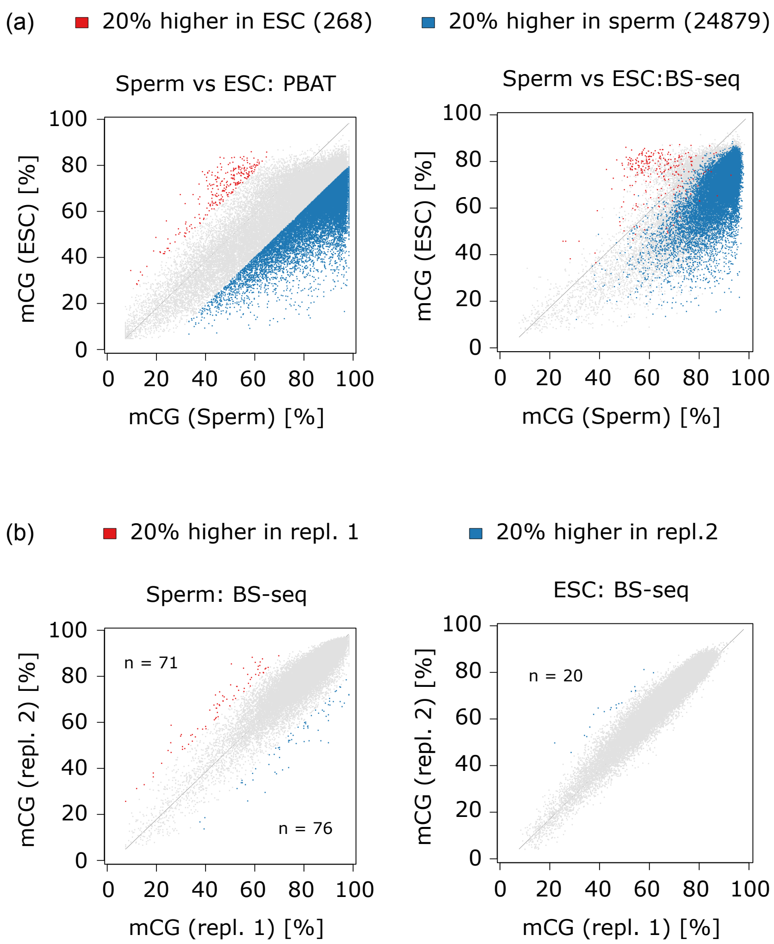

Figure S8. Relative methylation differences between differentially methylated regions. (**a**) Regions with over 20% mCG difference between sperm and mESC were selected in PBAT (left panel) and visualised for their positions in BS-seq datasets from the same samples (right panel). Averaged values were used for BS-seq (2 x ESC and 5 x sperm replicates) and a single replicate for PBAT. (**b**) Identified number of regions with over 20% mCG difference between two technical replicates from sperm and mESC in BS-seq datasets. Set 2 data (Kobayashi et al. 2012) was used in both (a) and (b).

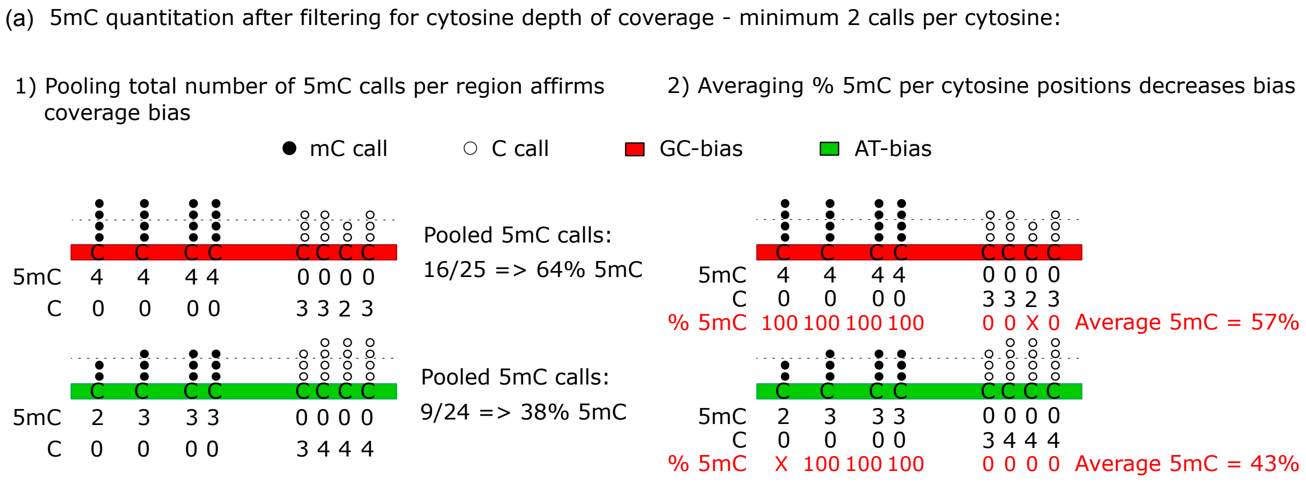

Figure S9. GC and AT coverage biases. (**a**) Comparison of the results from two methylation quantitation approaches, after filtering for coverage depth of at least 2 cytosine calls per position (dotted line).

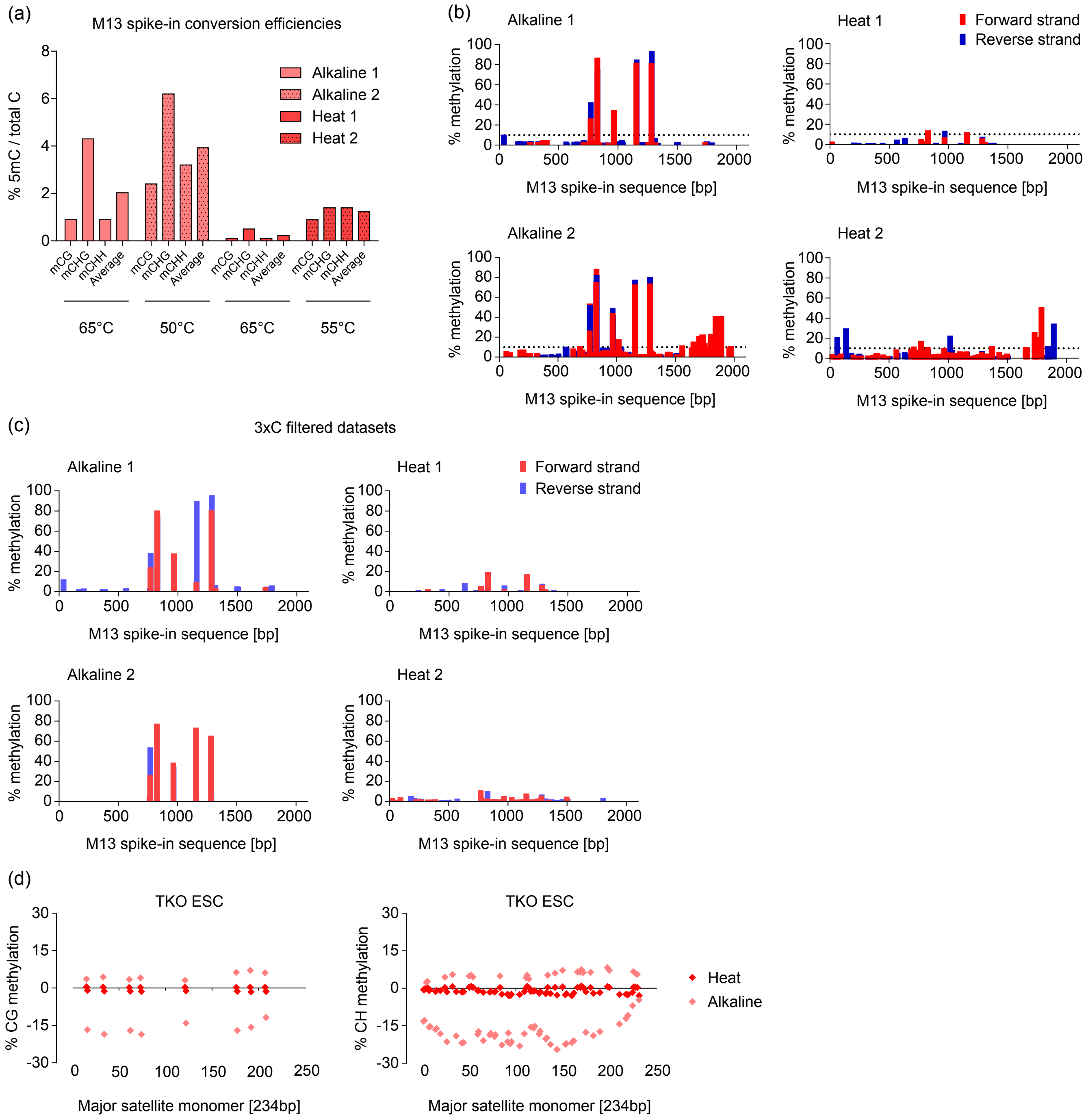

Figure S10. Incomplete conversion artefacts in BS-seq libraries prepared with alkaline and heat denaturation protocols. (**a**) Conversion of the unmethylated M13 spike-in DNA with four BS conversion protocols (see Table 1 for details on methods' parameters). (**b**) Incompletely converted and conversion resistant regions in the CHG context of the M13 spike-in sequence generated with four different conversion protocols. All sites resistant to conversion by the 'Alkaline' procedures are in CCWGG context, and they are successfully converted by the 'Heat' denaturation protocols. A dotted line shows the cut-off threshold at 10%, which can remove many but not all false positive calls, leaving a number of unconverted sites with moderately high or high 'methylation' values. (**c**) False positive calls remaining in the M13 spike-in sequence after applying bioinformatic filtering to remove every read containing three or more methylated cytosines in CH context. Most low and moderately unconverted values have been removed, apart from the CCWGG conversion resistant sites in the 'Alkaline' procedures. (**d**) Over-amplified unconverted cytosines in the CG and CH contexts of the unmethylated TKO mESC line, showing a clear bias towards the C-rich bottom strand. These position-specific 'baseline' conversion rates were subtracted from the methylation values in the WT mESC in Fig.6. Positive y-axis values indicate the top strand and negative – the bottom strand.
